# Elevated leptin and decreased adiponectin independently predict the post-thrombotic syndrome in obese and non-obese patients

**DOI:** 10.1038/s41598-018-25135-y

**Published:** 2018-05-02

**Authors:** Sandra Mrozinska, Joanna Cieslik, Elżbieta Broniatowska, Anetta Undas

**Affiliations:** 1 Department of Metabolic Diseases, Jagiellonian University Medical College, 15 Kopernika St., 31-501 Krakow, Poland; 2 University Hospital, 36 Kopernika St., 31-501 Krakow, Poland; 3 Department of Otolaryngology, Head and Neck Surgery, 5th Military Hospital with Polyclinic, 1-3 Wroclawska St., 30-901 Krakow, Poland; 4 Faculty of Medicine and Health Sciences, Andrzej Frycz Modrzewski Krakow University, 1 Gustawa Herlinga-Grudzińskiego St., 30-705 Krakow, Poland; 5 Krakow Centre for Medical Research and Technologies, John Paul II Hospital, 80 Pradnicka St., 31-202 Krakow, Poland; 6 Institute of Cardiology, Jagiellonian University Medical College, 80 Pradnicka St., 31-202 Krakow, Poland

## Abstract

Post-thrombotic syndrome (PTS) is a common complication of deep vein thrombosis (DVT). Little is known about the involvement of adipokines in the pathogenesis of DVT. We evaluated whether adipokines can predict PTS. In a prospective cohort study, 320 DVT patients aged 70 years or less were enrolled. Serum adiponectin, leptin and resistin levels were measured three months since the index first-ever DVT. After 2 years’ follow-up PTS was diagnosed in 83 of 309 available patients (26.9%) who had 13.9% lower adiponectin and 16% higher leptin levels compared with the remainder (both p < 0.0001). No PTS-associated differences in C-reactive protein, fibrinogen, D-dimer, plasminogen activator inhibitor-1 and resistin were observed. The multivariable logistic regression adjusted for age, sex, obesity and tissue plasminogen activator (tPa) showed that lower adiponectin (odds ratio [OR], 0.42; 95% confidence interval [CI], 0.31–0.56) and higher leptin levels (OR, 1.49; 95% CI, 1.31–1.69) are independent predictors for PTS. Obesity-stratified logistic regression analysis confirmed that lower adiponectin (OR, 0.49; 95% CI, 0.38–0.64) and higher leptin (OR, 1.41; 95% Cl, 1.25–1.58) levels predicted PTS. Our findings showed that lower adiponectin and higher leptin measured 3 months after DVT, regardless of obesity, can independently predict PTS, which suggests novel links between adipokines and thrombosis.

## Introduction

Post-thrombotic syndrome (PTS) is a common complication of deep vein thrombosis (DVT)^1^, that manifests as pain, a sensation of swelling, cramps, heaviness, edema, redness and telangiectasia in the lower limbs^1,2^. PTS develops in 20–50% patients after DVT^2–4^ and increases a risk of recurrent venous thromboembolism (VTE)^5^. Other factors predisposing to VTE recurrence include proximal unprovoked DVT, first symptomatic pulmonary embolism (PE), severe thrombophilia, and several emerging biomarkers^6–9^. The well-established risk factors for PTS are obesity, proximal DVT, and ipsilateral DVT recurrence^10^.

There is evidence that inflammation is involved in the pathogenesis of PTS^11,12^. Elevated inflammatory markers, including interleukin (IL)-10, C-reactive protein (CRP), and IL-6, have been reported in patients with PTS^11–13^.

Obesity, a risk factor for PTS^2,10,14,15^, is associated with a pro-inflammatory and prothrombotic state^16,17^. The adipose tissue secretes several hormones and cytokines known as adipokines, including IL-6, tumor necrosis factor-alpha (TNF-α), leptin, adiponectin, plasminogen activator inhibitor 1 (PAI-1), resistin, and others^18,19^. Adiponectin has been reported to have antithrombotic properties, while leptin is considered to have a prothrombotic effect^20^. *In vitro* studies showed that leptin promoted platelet aggregation^21^ and enhanced PAI-1, but not tissue-type plasminogen activator (tPa) expression in endothelial cells^22^. Animal studies showed that leptin is associated with venous thrombosis and PE^23^, while adiponectin decreased platelet aggregation by promoting endothelial nitric oxide and attenuate oxidative/nitrative stress^24^. Lower adiponectin levels were observed among patients with confirmed PE^25^. Allison *et al*.^26^ reported that adipokines, i.e. resistin, leptin, TNF-α, are associated, independently of obesity and waist circumstance, with chronic venous disease.

To our knowledge, there have been no reports exploring the association between adipokines and PTS. Given links of obesity to PTS^2,10,14,15^ and experimental data on the impact of adipokines on prothrombotic mechanisms in various models^21–26^, we conducted a prospective cohort study to assess a predictive value of resistin, adiponectin and leptin in the PTS development.

## Methods

### Patients

We screened 368 consecutive adult patients aged 70 years or less, after first-ever DVT alone or combined with PE. We measured adipokines levels in one point time, 3 months since DVT and assessed the presence of PTS after a 2 years’ follow-up. The patients were referred to the outpatient clinic for further laboratory work-up between October 2008 and June 2012. The subgroup of this population was described in detail in our previous paper^27^. The diagnosis of DVT was established by a positive finding of color duplex sonography (visualization of an intraluminal thrombus in calf, popliteal, femoral, or iliac veins). The diagnosis of PE was based on the presence of typical symptoms and positive results of high resolution spiral computed tomography (CT), at least one subsegmental filling defect.

The exclusion criteria and flow chart of the study design can be found in Supplementary Fig. 1.

All patients received anticoagulant therapy with unfractionated or low-molecular-weight heparins, subsequently vitamin K antagonists (VKA) were implemented. The duration of the treatment was 3 months or more in patients with provoked VTE, or 6 months or longer in patients with unprovoked VTE at the discretion of the treating physicians. Compression stockings class II or hosiery were prescribed to all patients for 6–24 months at the time of DVT diagnosis.

Unprovoked (idiopathic) VTE episode was defined as not having surgery requiring general anesthesia, major trauma, plaster cast or hospitalization in the last month, taking oral contraceptives, pregnancy or delivery in the last 3 months. A proximal DVT event was defined as thrombosis in the popliteal vein, including the trifurcation, femoral or iliac veins. Positive family history of VTE was defined as a history of documented VTE in at least one first degree relative. Obesity was defined as BMI ≥30 kg m^−2^.

The study was approved by the Jagiellonian University Bioethical Committee. All the patients gave written informed consent to participate in the study. The research was conducted in accordance with the Declaration of Helsinki.

### Follow-up

The primary endpoint of the study was the presence of PTS after a 2 years’ follow-up based on the Villalta scale^1^. The study participants were examined after 18 to 24 months at the clinic. The subjects suspected of PTS were re-evaluated on the second visit after 3 to 6 months and the final score was analyzed. Evaluation was performed by one physician, blinded to the results of laboratory investigations or duplex ultrasound. The Villalta scale contains eleven symptoms and clinical signs: pain, cramps, heaviness, paresthesia, pruritus, pretibial edema, skin induration, hyperpigmentation, redness, venous ectasia, pain on calf compression and venous ulcer. The points are given from 0 to 3 depending on the severity of the above features. PTS was diagnosed based on 5 points or more on two consecutive visits, at least 3 months apart. The severity of PTS was classified as mild (5 to 9 points), moderate (10 to 14 points) or severe (>14 points)^1^.

VTE recurrences were recorded and confirmed with imaging, i.e. color duplex sonography for DVT and high resolution spiral CT for PE. In cases of suspected DVT recurrence in the same leg as the index event, non-compressibility of a previously compressible venous segment or an increase of at least 4 mm in the residual diameters were applied to confirm the diagnosis. Following a recurrent thromboembolic event patients received a low-molecular-weight heparin, usually enoxaparin, at therapeutic doses with the subsequent VKA administration and the evaluation for PTS was performed 2 years after the index DVT.

We collected data on the medications used, especially the duration of VKA anticoagulation; subjects who stopped anticoagulation, in particular those after unprovoked events, were advised to use sulodexide 250–500 mg bid and/or aspirin 75–100 mg once daily. None of the patients was treated with direct oral anticoagulants during follow-up. Patients who declared regular use of compression therapy for a period of 6–24 months (usually with a 6–8 weeks’ interruption in the June-August weeks) were recorded.

### Laboratory investigation

After 3 months (±2 weeks) of anticoagulant therapy after DVT, fasting blood samples were taken from an antecubital vein with minimal stasis between 8.00 and 11.00 AM as described previously^27^. Patients treated with VKA were switched to a low-molecular-weight heparin for 10–14 days before and blood samples were collected after 16–24 hours after the last injection, subsequently the anticoagulation with VKA was continued. High-sensitivity CRP was assessed using immunoturbidimetry (Roche Diagnostics, Mannheim, Germany). Fibrinogen was measured with the Clauss assay. Plasma D-dimer was determined with the Innovance D-dimer assay (Siemens, Marburg, Germany). Leptin, adiponectin and resistin were measured in serum with the Bio-Rad Luminex flow cytometry (Millepore, Billerica, MA, USA). Leptin, adiponectin and resistin levels in age- and BMI- matched control subjects free of VTE (n = 47, 27 women, aged 26–64 years) assayed in our lab served as comparators for adipokines in the patient cohort.

PAI-1 and tPa antigens were determined in citrated plasma using immunoenzymatic assays (both American Diagnostica, Stamford, CT, USA). All patients were screened for thrombophilia as described^28^.

Plasma thrombogenic potential was assessed using calibrated automated thrombography (CAT) (Thrombinoscope BV, Maastricht, the Netherlands) in a 96-well plate fluorometer (Ascent Reader, Thermolab systems OY, Helsinki, Finland) at 37 °C according to the manufacturer’s instructions as described^29^. Briefly, 80 microliters of platelet poor plasma were diluted with 20 µL of a tissue factor-based activator (Diagnostica Stago, Asniéres, France) containing 5 pmol/L recombinant tissue factor, 4 micromolar phosphatidylserine/phosphatidylcholine/phosphatidylethanolamine vesicles, and 20 µL of FluCa solution (Hepes, pH 7.35, 100 nmol/L CaCl_2_, 60 mg/mL bovine albumin, and 2.5 mmol/L Z-Gly-Gly-Arg-amidometylcoumarin). The peak thrombin level was analyzed in duplicate. Intraassay variability was 6%.

### Statistical analysis

The study was powered to have a 80% chance of detecting a difference in adiponectin levels using an alpha value of 0.05, based on the values of adiponectin presented in a previous study^25^. Taking into account that about 20% of patients develops PTS, in order to demonstrate such a difference or greater, at least 166 patients were required in the control group and 42 patients in the PTS group. The Cohen’s effect size was 0.5. The calculations were performed using G* Power Version 3.0.10^30^.

The continuous variables are presented as mean ± standard deviation (or median and interquartile range) whereas the categorical and qualitative variables are expressed as the number (percentages). The normality of variable distribution was verified by the Shapiro-Wilk test. The Levene’s test was used to assess the equality of variances. The parametric Student *t*-test was used for continuous variables if the criteria for normality and equality of variances were fulfilled. Otherwise the Mann-Whitney *U* or the Welch tests were performed. Categorical variables were compared between groups using the chi-square test or Fisher’s exact test. The Spearman’s rank correlation test was used to explore the association between two continuous variables. Both univariate and multivariable logistic regression models were calculated to establish the risk factors for PTS. The linear association of all continuous predictors with PTS was assessed using Generalized Additive Models with the LOWESS curve smoothing method. Multivariable logistic model was fitted using backward stepwise regression. The Akaike information criterion (AIC) was applied to compare multivariable logistic regression models. The p-value < 0.25 was selected as the threshold for including variables in the multivariable logistic model. Moreover, potential and known confounders and risk factors (like age, sex, obesity/BMI, proximal DVT, ipsilateral recurrent DVT, tPa) were considered in the studied models. The C-statistic (i.e. area under the curve [AUC]) was used to assess whether adipokines contributed significantly to predictive ability of PTS. The determination of the better model was done by the pair-wise comparison of C-statistic values with Bonferroni correction (significance level of 0.017 was applied). The obesity-stratified regression model was studied in order to assess the predictive value of adipokines independently of obesity. The receiver operating characteristic (ROC) curves were calculated to determine the optimal cut-off levels of adipokines as predictors of PTS. The AUC was also analyzed. The p-values < 0.05 were considered statistically significant for two-sided tests. The calculations were performed using Statistica 12.5 software (StatSoft Inc., Tulsa, Oklahoma, United States) and R package^31^.

### Data Availability

The datasets analysed during the current study are available from the corresponding author on reasonable request.

## Results

A total of 309 patients at a median age of 46 years were included in the final analysis (Table 1, Supplementary Fig. 1, Supplementary Table 1). The minimum age was 18 and the maximum age was 69 years. Eleven patients were lost to follow-up. Most of the patients had isolated, unprovoked DVT. As expected, obese subjects (n = 63, 20.4%) had slightly higher leptin and lower adiponectin levels compared with those with BMI < 30 kg m^−2^ (n = 246, 79.6%, Supplementary Table 2). There were no gender- or age-related differences in adipokines (data not shown).

**Table 1:** Patient Characteristics.

Variable	All patients (n = 309)	No PTS (n = 226)	PTS (n = 83)	P value
Age, years	46 (36–54)	47 (37–53)	44 (33–56)	0.36
Male sex, n (%)	150 (48.5)	104 (46.0)	46 (55.4)	0.14
BMI, kg m^−2^	26.1 (23.7–29.4)	25.7 (23–29)	27.3 (25–29.8)	0.002
Obesity, n (%)	63 (20.4)	43 (19)	20 (24.1)	0.34
Duration of anticoagulation treatment, months	10 (7–12)	10 (7–12)	10 (7–12)	0.55
**Clinical characteristics, n (%)**				
Unprovoked VTE	157 (50.8)	112 (49.6)	45 (54.2)	0.47
DVT alone	242 (78.3)	177 (78.3)	65 (78.3)	1.00
Localization of isolated DVT, n (%)				
proximal DVT	171 (55.3)	123 (54.4)	48 (57.8)	0.59
distal DVT	71 (23)	54 (23.9)	17 (20.5)	0.53
PE + DVT	67 (21.7)	49 (21.7)	18 (21.7)	1.00
Proximal DVT	238 (77)	172 (76)	66 (79.5)	0.53
Family history of VTE	46 (14.9)	30 (13.3)	16 (19.3)	0.19
Compression therapy	211 (68.3)	158 (69.9)	53 (63.9)	0.31
**Laboratory investigations**				
CRP, mg L^−1^	1.5 (0.9–2.3)	1.5 (0.9–2.2)	1.7 (0.9–2.3)	0.42
INR	0.98 (0.9–1.04)	0.98 (0.91–1.04)	0.97 (0.87–1.04)	0.14
Fibrinogen, g L^−1^	3.03 (2.53–3.86)	3.02 (2.52–3.81)	3.04 (2.53–4.04)	0.70
D-dimer, ng mL^−1^	279 (226–337)	277 (226–337)	287 (221–347)	0.89
tPa, ng mL^−1^	9.6 (7.2–11.4)	9.4 (6.9–11.2)	10.3 (8–11.9)	0.02
PAI-1, ng mL^−1^	11.2 (8.5–14.7)	11.1 (8.5–13.9)	11.9 (8.5–15.0)	0.18
Peak thrombin, nM	245.7 (210–291.1)	244.8 (210–292.5)	246.2 (210–286.4)	0.93
**Adipokines**				
Adiponectin, µg mL^−1^	14.9 (13.7–16.8)	15.8 (14.2–17.7)	13.6 (12.5–14.7)	<0.0001
Leptin, ng mL^−1^	27.5 (24.4–30.3)	26.2 (22.8–28.4)	30.4 (28.8–32.8)	<0.0001
Resistin, pg mL^−1^	14.9 (13.9–16.2)*	14.8 (13.8–16.2)*	15.2 (14–16.5)	0.38

Values are given as mean ± standard deviation (SD), median (interquartile range) or number (percentage).

PTS, post-thrombotic syndrome; BMI, body mass index; VTE, venous thromboembolism; DVT, deep vein thrombosis; PE, pulmonary embolism; CRP, C-reactive protein; INR, international normalized ratio; tPa, tissue plasminogen activator; PAI-1, plasminogen activator inhibitor-1. *Data unavailable for one patient.

We compared the DVT patient and controls matched for age and BMI, the former group had lower adiponectin (14.9 [13.7–16.8] versus 18.6 [17.5–21.4] µg mL^−1^, p < 0.001) and higher leptin (27.5 [24.4–30.3] versus 15.3 [14.2–17.2] ng mL^−1^, p < 0.001) levels, while there was no difference in resistin concentrations (14.9 [13.9–16.2] versus 14.7 [13.5–15.9] pg mL^−1^, p = 0.27), which is in line with the study by Allison *et al*.^26^.

Men with DVT who were younger than control men (45 [36–55] vs 55.5 [41–61] years; p = 0.01) had lower adiponectin (14.9 [13.6–17] vs 18.5 [17.7–20.5] µg mL^−1^) and higher leptin 27.4 [24.8–30.1] vs 15.1 [14.2–17.2] ng mL^−1^; all p < 0.001, respectively). Also women with DVT when compared with control women had lower adiponectin (14.8 [13.7–16.6] vs 19.5 [17.5–21.5] µg mL^−1^) and higher leptin (27.7 [24.2–30.4] vs 15.4 [14.3–16.8] ng mL^−1^; p < 0.001, respectively) when analyzed separately.

Interestingly, patients with unprovoked DVT compared with those with provoked DVT had slightly higher leptin (27.9 [24.9–30.4] versus 26.9 [23.7–30] ng mL^−1^, p = 0.04), lower resistin (14.7 [13.7–15.9] versus 15.2 [14.0–16.7] pg mL^−1^, p = 0.02), and tended to have lower adiponectin concentrations (14.7 [13.5–16.6] versus 15 [13.9–16.9] µg mL^−1^, p = 0.06, respectively) despite similar BMI values in the two subgroups (26.1 [23.8–29.3] versus 26 [23.4–29.4] kg m^−2^, p = 0.72). Adipokines did not differ between patients with isolated DVT versus those with DVT combined with PE as well as between patients with proximal DVT versus distal DVT (data not shown).

**Figure 1:**
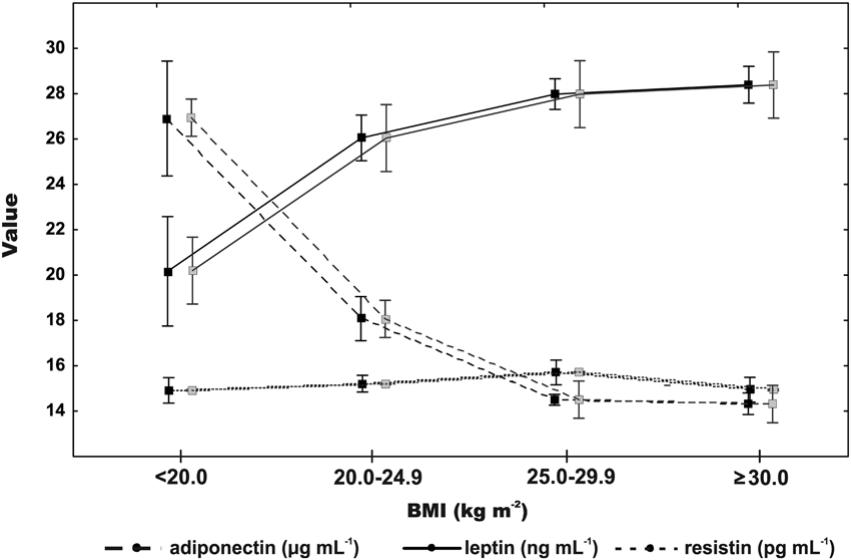
Mean adipokines levels (adiponectin, leptin, resistin) non-adjusted (black small boxes) and adjusted (grey small boxes) for sex and age across four body mass index categories. Boxes represent mean values, “whiskers” represent a 95% confidence interval (CI). The CIs in resistin levels are very narrow, so that are hardly visible.

Adiponectin was inversely correlated with leptin (r = −0.45, p < 0.0001). Resistin was weakly correlated with leptin (r = 0.13, p = 0.03), but not with adiponectin (p = 0.28). As expected, there was a positive association of BMI and leptin (r = 0.33, p < 0.0001) and a negative correlation with adiponectin (r = −0.52, p < 0.0001). Mean adipokines levels adjusted for age and gender across four BMI categories are shown in Fig. 1.

There were no associations between adipokines and peak thrombin, fibrinogen, CRP, and D-dimer. Resistin and leptin, but not adiponectin, were positively correlated with tPa (r = 0.32, p < 0.0001; and r = 0.17, p = 0.003) and PAI-1 antigens (r = 0.34, p < 0.0001; and r = 0.14, p = 0.014, respectively).

After 2 years’ follow-up PTS was diagnosed in 83 (26.9%) patients, including mild PTS in 37 (44.6%), moderate PTS in 30 (36.1%), and severe PTS in 16 (19.3%) individuals. There were no leg ulcers among patients. There was no difference in age, sex and the duration of anticoagulant treatment between the PTS and non-PTS group. PTS was associated with slightly greater BMI, higher triglycerides and tPa levels (Table 1, Supplementary Table 1). There were no differences in proportions of patients who discontinued anticoagulation after 3–6 months as well as after 6–12 months since DVT between the PTS and non-PTS groups (data not shown).

Patients who developed PTS had 13.9% lower adiponectin and 16% higher leptin levels measured 3 months after DVT when compared with those free of this complication, with no difference in resistin concentrations (Table 1). A similar trend was observed in non-obese patients, i.e.13.1% lower adiponectin (p < 0.001) and 20% higher leptin (p < 0.001) when compared individuals with PTS versus those without PTS. This was also true for obese subjects with PTS, who had 17.4% lower adiponectin (p < 0.001) and 14.3% higher leptin (p < 0.001) compared with those free of this complication.

**Figure 2:**
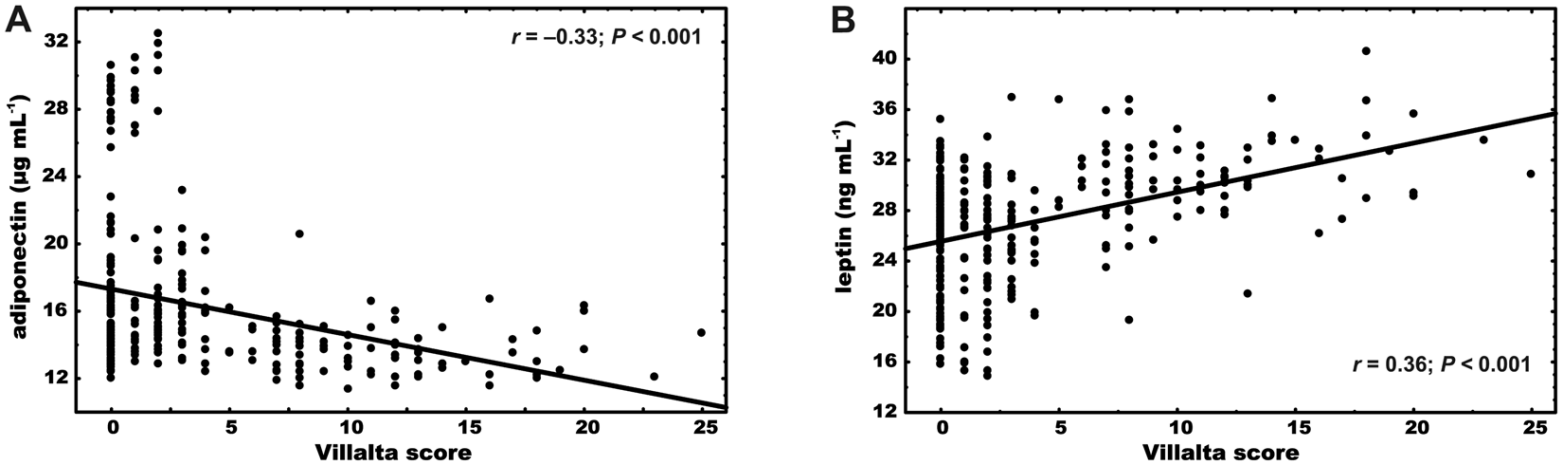
Correlations between the Villalta scores and two adipokines. The associations between variables measured 3 months since VTE in 309 patients - (**A**) adiponectin, (**B**) leptin and the Villalta score assessed after a two-year follow-up since venous thromboembolism (VTE) episodes.

In the whole cohort leptin and adiponectin were correlated with the Villalta score (r = 0.36, p < 0.0001, and r = −0.33, p < 0.0001, respectively, Fig. 2). In subjects diagnosed with PTS there were no such associations. There were no differences in adiponectin (13.6 [12.7–14.6] versus 13.3 [12.2–14.8] µg mL^−1^, p = 0.66), leptin (30.3 [28.3–32.2] versus 32.4 [29.3–33.8] ng mL^−1^, p = 0.08) and resistin levels (15.2 [14–16.5] versus 15 [14.2–16.5] pg mL^−1^, p = 0.92) between the non-severe and severe PTS subgroups.

The presence of PTS was associated with higher BMI, higher leptin, and lower adiponectin levels (all p < 0.05; Table 2 and Supplementary Table 3). The multivariable logistic regression model adjusted for age, sex, obesity and tPa showed that higher leptin and lower adiponectin levels are independent predictors for PTS in this DVT patient cohort (Table 2). The C-statistic for this regression model was 0.9, whereas in the case of the same model without adipokines, it was 0.63 (p < 0.0001, for comparison of both models). In the model including BMI as a continuous variable instead of obesity with adjustment for age, sex, BMI and tPa, elevated leptin and decreased adiponectin also predicted PTS and the C-statistic was 0.9 (Supplementary Table 3). The C-statistic for the same model without adipokines was 0.68 (p < 0.0001, for comparison of both models).

**Table 2:** The logistic Regression Model for Risk Factors of PTS.

Variable	OR per	Univariate		Multivariable*	
		OR (95% CI)	P	OR (95% CI)	P
Age^†^	10 years	0.89 (0.72–1.10)	0.27		
Male^†^	No/Yes	1.46 (0.88–2.42)	0.14	1.83 (0.91–3.66)	0.09
Obesity^†^	No/Yes	1.35 (0.74–2.47)	0.33	0.61 (0.26–1.42)	0.25
Duration of anticoagulation treatment	1 month	1.03 (0.96–1.10)	0.49		
Smoking	No/Yes	0.94 (0.56–1.60)	0.83		
Unprovoked VTE	No/Yes	1.21 (0.73–2.00)	0.47		
Proximal DVT	No/Yes	1.22 (0.66–2.25)	0.53		
Recurrent DVT ipsilateral	No/Yes	1.51 (0.58–3.92)	0.4		
Family history of VTE	No/Yes	1.56 (0.80–3.04)	0.19		
Compression therapy	No/Yes	0.76 (0.45–1.30)	0.31		
Aspirin^‡^	No/Yes	0.71 (0.33–1.56)	0.39		
Sulodexide^‡^	No/Yes	1.18 (0.55–2.50)	0.68		
Statins	No/Yes	1.08 (0.65–1.80)	0.76		
Creatinine	1 µmol L^−1^	1.01 (0.99–1.03)	0.22		
Glucose	1 mmol L^−1^	1.05 (0.73–1.52)	0.79		
Triglycerides	1 mmol L^−1^	1.36 (0.94–1.97)	0.11		
CRP	1 mg L^−1^	1.07 (0.90–1.27)	0.43		
Fibrinogen	1 g L^−1^	1.08 (0.82–1.42)	0.59		
D-dimer	100 ng mL^−1^	1.01 (0.78–1.31)	0.91		
tPa^†^	1 ng mL^−1^	1.11 (1.02–1.21)	0.02	1.06 (0.95–1.2)	0.31
PAI-1	1 ng mL^−1^	1.04 (0.99–1.08)	0.08		
Peak thrombin	10 nM	0.99 (0.96–1.03)	0.77		
Adiponectin	1 µg mL^−1^	0.52 (0.43–0.64)	<0.001	0.42 (0.31–0.56)	<0.0001
Leptin	1 ng mL^−1^	1.41 (1.28–1.56)	<0.001	1.49 (1.31–1.69)	<0.0001
Resistin	1 pg mL^−1^	1.02 (0.92–1.12)	0.75		

OR, odds ratio; CI, confidence intervals; other abbreviations see Table 1. ^*^Multivariable model was fitted using backward stepwise regression. Adjusted for age. ^†^Variable locked in the model. ^‡^Medications initiated after anticoagulation withdrawal during follow-up.

Importantly, the obesity-stratified logistic regression analysis adjusted for age, sex and tPa also confirmed that lower adiponectin (OR, 0.49; 95% CI, 0.38–0.64) and higher leptin (OR, 1.41; 95% Cl, 1.25–1.58) levels were predictors for PTS (Supplementary Table 4).

Models including a single adipokine were built and also showed that lower adiponectin and higher leptin are risk factors for PTS independently of obesity (data not shown).The C-statistic for the model with only adiponectin was 0.84 (p < 0.001, for the comparison with the model without adipokines), whereas C-statistic for the model with only leptin was 0.83 (p < 0.001, for the comparison with the model without adipokines).

**Figure 3:**
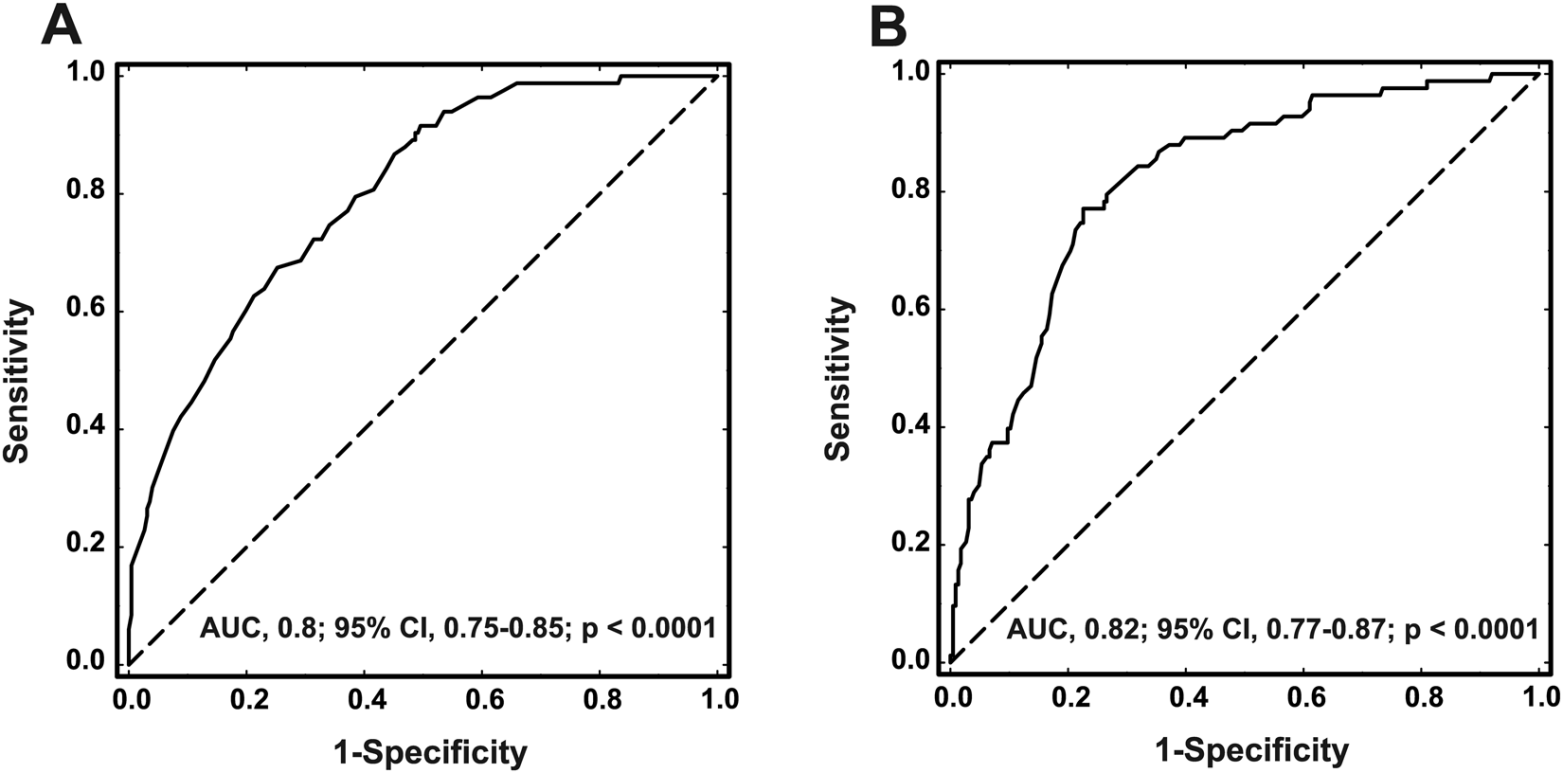
Receiver operator characteristic (ROC) curve analysis to test the ability of adiponectin (**A**) and leptin (**B**) to identify patients who developed PTS (n = 83) during two-year follow-up. Optimal cutoffs and area under the curve (AUC) were of 0.8 (95% confidence interval [CI], 0.75–0.85; cutoff, 12.8 µg ml^−1^) for adiponectin and 0.82 (95% CI, 0.77–0.87; cutoff, 32.1 ng ml^−1^) for leptin.

ROC analysis showed moderate specificity for adiponectin and leptin to predict PTS development using optimal cutoffs with AUC of 0.8 (95% confidence interval [CI], 0.75–0.85; cutoff, 12.8 µg mL^−1^) and 0.82 (95% CI, 0.77–0.87; cutoff, 32.1 ng mL^−1^, respectively, Table 3 and Fig. 3). Univariate logistic regression for categorical variables based on optimal cut-offs showed that leptin >32.1 ng mL^−1^ and adiponectin ≤12.8 µg mL^−1^ are associated with PTS occurrence during follow-up (Supplementary Table 5). The multivariable logistic regression model adjusted for age, sex, obesity, and tPa showed that increased leptin (values >32.1 ng mL^−1^) and decreased adiponectin (values ≤12.8 µg mL^−1^) levels predicted PTS (Supplementary Table 5). The C-statistic for the model was 0.76.

**Table 3:** Measures of Performance of Adipokines to Predict the PTS.

Variable	Sensitivity (95%CI)	Specificity (95%CI)	PPV (95%CI)	NPV (95%CI)	LR positive (95%CI)	OR (95%CI)
Adiponectin 12.8 µg mL^−1^	0.30 (0.21–0.41)	0.96 (0.92–0.98)	0.74 (0.56–0.87)	0.79 (0.74–0.84)	7.56 (3.68–15.53)	10.39 (4.60–23.48)
Leptin 32.1 ng mL^−1^	0.34 (0.24–0.45)	0.95 (0.91–0.97)	0.7 (0.53–0.83)	0.8 (0.74–0.84)	6.35 (3.39–11.9)	9.08 (4.34–19.00)

Based on the optimal cut-off levels of adipokines as predictors of PTS that were determined based on the receiver operating characteristic (ROC) curves.

PPV, positive predictive value; NPV, negative predictive value; LR, likelihood ratio; other abbreviations see Tables 1 and 2.

Recurrent DVT was observed in 35 (11.3%) individuals during 2 years’ follow-up, including 17 cases during anticoagulation treatment. There was no difference between the PTS and non-PTS group regarding the recurrent VTE (Supplementary Table 6).

The analysis of the patients after exclusion of those with recurrent DVT during follow-up after adjustment for age, sex, obesity and tPa also showed that decreased adiponectin (OR, 0.44; 95% CI, 0.32–0.63) and increased leptin (OR, 1.46; 95% CI, 1.26–1.69) predicted PTS in patients free of VTE recurrences.

## Discussion

This study is the first to show the association between adipokines and the development of PTS after a 2-years’ follow-up in a cohort of young and middle-aged DVT patients. We observed that elevated leptin and decreased adiponectin levels measured in one point time, 3 months since the first-ever DVT episode are independent predictors of PTS regardless of inflammatory state and obesity. Given the paucity of clinical data on the impact of circulating adipokines on thrombosis, our findings extend the current knowledge of adipokines in thrombosis in humans.

There are accumulating data largely derived from *in vitro* studies and animal models to show that adipokines are involved in thrombosis. It has been demonstrated that administration of leptin neutralizing antibodies was associated with protection from lethal venous thrombosis and PE^23^. A similar effect was observed in leptin deficient ob/ob mice^23^. Dellas *et al*. showed that among 264 patients with acute PE lower leptin levels at admission predicted death, circulatory collapse with need for catecholamines, intubation or resuscitation, in a 30-day follow-up^32^. No data regarding a prognostic value of leptin among DVT patients have been published yet. Adiponectin is suggested to have anti-aggregatory properties^24,33^. Patients with confirmed PE had lower adiponectin compared with the control group^25^.

Our study expanded observations made by Allison *et al*.^26^ in 2010. They demonstrated that resistin, leptin, and TNF-α are associated, independently of obesity and waist circumstance, with chronic venous disease. However, in contrast to our study, the Villalta score was not used by Allison *et al*.^26^ and previous DVT was not presented in association with adipokines. In the former study, increased intra-abdominal pressure, and multiple inflammatory/metabolic effects of adipokines were postulated to contribute to venous disease^26^. We found that inflammatory mechanisms are rather unlikely to largely contribute to PTS, however, this issue requires further study.

Given strong links between body weight and adipokines, our findings are important since the predictive value of adiponectin and leptin for PTS remained significant after adjustment for BMI as well as obesity. Moreover, the obesity-stratified logistic regression analysis showed that lower adiponectin and higher leptin predict PTS suggesting that their harmful effect is independent of obesity. Whether weight reduction lowers the risk of PTS also through changes in unfavorable adipokine profiles, deserves further investigation.

We did not notice differences in adipokines levels related to gender and age in our DVT cohort. However, there are inconclusive reports regarding age- and sex- related differences in adipokines, and this issue merits further investigagtion^34,35^.

Enhanced inflammation reflected by elevated CRP and IL-10 has been shown to be risk factor for developing PTS^11,12^. We failed to show higher CRP at 3 months in patients who developed PTS after 2 years since the index DVT. However, we excluded patients with CRP above 15 mg L^−1^. Roumen-Klappe *et al*. found a weak association between CRP and PTS diagnosed according to the Clinical Etiologic Anatomic Pathologic classification, and no association with the Villalta-defined PTS^36^; the scale used in our report. Other studies, like ours, also did not find any relation between CRP levels and PTS development^12^. We cannot exclude that other inflammatory markers are better predictors of PTS, however, assessment of interleukins or adhesion molecules was beyond the scope of this study.

Mechanisms underlying an elevated risk of PTS in subjects with higher leptin and lower adiponectin are likely multifactorial^20–26,32^. In our study there was a weak positive correlation between leptin and PAI-1, which agrees with a recent study on healthy and hypertensive individuals^37^. We may hypothesize that hypofibrinolytic effects of disturbed adipokines profiles in circulating blood contribute to our observations. Given the data showing an increased risk of PTS in patients with prolonged clot lysis time^27^, the current findings might support the association between hypofibrinolysis and PTS. Moreover, adipokines could be linked with chronic venous disease by their metabolic effect on insulin resistance involved in thrombosis^38,39^.

Several study limitations should be acknowledged. First, our study was relatively small but adequately powered to show an expected rate of PTS during follow-up similar to that reported in the literature^2,14^. We studied relatively young DVT patients, so the current results cannot be easily extrapolated to all DVT patients, especially to the elderly with several comorbidities. Laboratory variables including adipokines were measured in a single point time, therefore changes with time in some of them cannot be excluded for example due to weight increase or decrease. However, most of the patients reported their weight difference at 2 years at a level of about 5% compared with the initial values. Some patients stopped anticoagulation after a few months even if they suffered from proximal unprovoked DVT, so it is unclear whether prolonged treatment or the use of direct oral anticoagulants since the diagnosis of DVT may affect the risk of PTS and its association with adipokines^40^. Elucidation of the precise molecular mechanisms behind the observed impact of adipokines on PTS was beyond the scope of the present study and should be explored in the future.

In conclusion, our study showed that higher leptin and lower adiponectin predicted PTS in relatively young DVT patients regardless of obesity. This suggests previously unknown links of adipokines and thrombosis and its complications. If future studies confirm a predictive value of elevated leptin and decreased adiponectin for PTS it could help identify patients at increased risk of PTS development, who need an individualized treatment.

## Electronic supplementary material

Supplementary Information
