# Supplementary material for: Elevated leptin and decreased adiponectin independently predict the post-thrombotic syndrome in obese and non-obese patients
Source: Sci Rep. 2018 May 2;8:6938. doi: 10.1038/s41598-018-25135-y (PMC5932041; doi:10.1038/s41598-018-25135-y)
Supplement: Supplementary file 1 — Supplementary Information [file 41598_2018_25135_MOESM1_ESM.pdf]

Elevated leptin and decreased adiponectin independently predict the post-thrombotic syndrome in obese and non-obese patients

The running title: Adipokines and post-thrombotic syndrome

Sandra Mrozinska,[1,2] Joanna Cieslik,[3] Elżbieta Broniatowska,[4] and Anetta Undas[5,6]

[1]Department of Metabolic Diseases, Jagiellonian University Medical College, 15 Kopernika St., 31-501 Krakow, Poland;
[2]University Hospital, 36 Kopernika St., 31-501 Krakow, Poland;
[3]Department of Otolaryngology, Head and Neck Surgery, 5th Military Hospital with Polyclinic, 1-3 Wroclawska St., 30-901 Krakow, Poland;
[4]Department of Bioinformatics and Telemedicine Jagiellonian University Medical College, 16 Sw. Lazarza St., Krakow, Poland;
[5]Krakow Centre for Medical Research and Technologies, John Paul II Hospital, Krakow, Poland;
[6]Institute of Cardiology, Jagiellonian University Medical College, 80 Pradnicka St., 31-292 Krakow, Poland

Supplementary Information

Supplementary Figure 1. Flow chart of the study design and the participant recruitment.

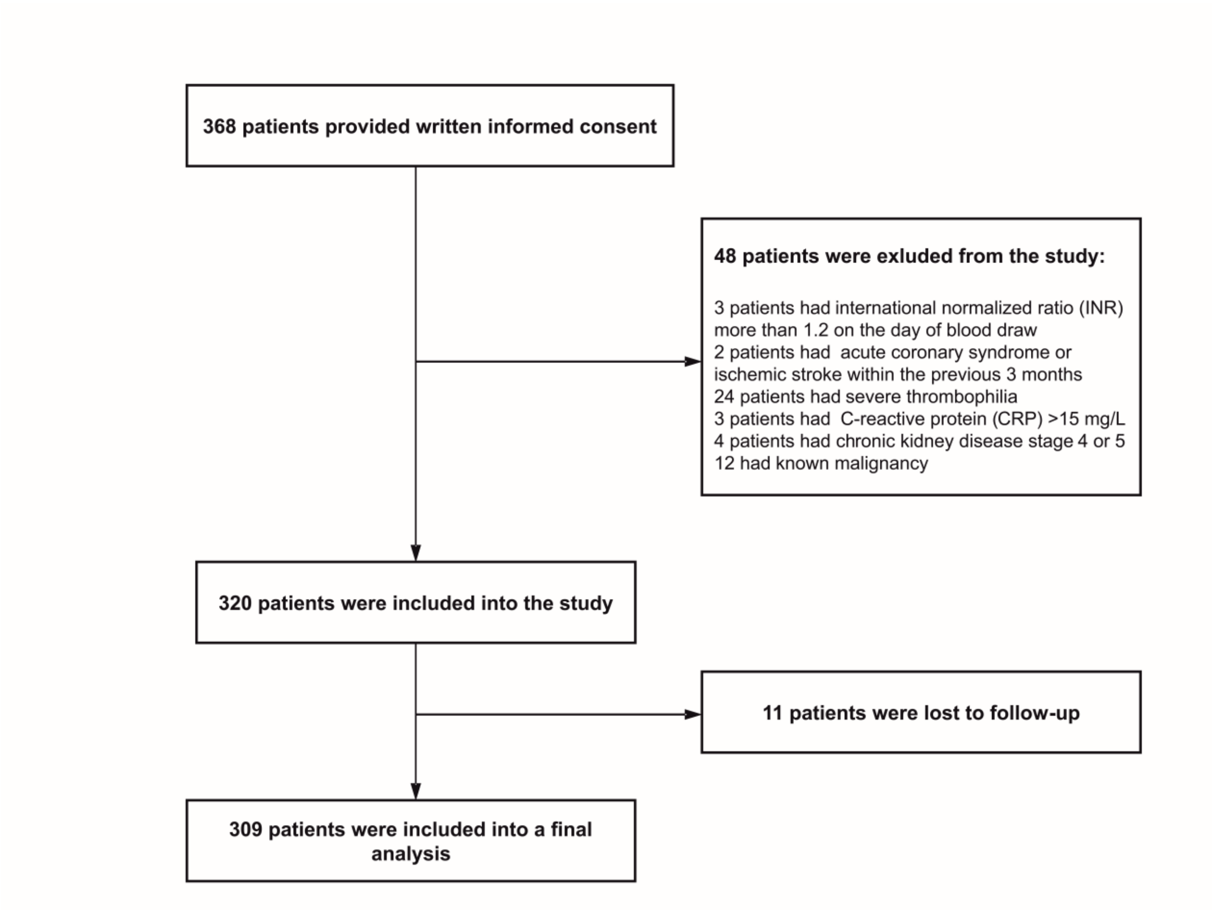

Supplementary Table 1. Additional patient characteristics.

Variable	All patients (n=309)	No PTS (n=226)	PTS (n=83)	P value
Clinical characteristics, n (%)				
Current smokers	111(35.9)	82 (36.3)	29 (34.9)	0.83
Trauma or surgery	87 (28.2)	66 (29.2)	21 (25.3)	0.5

Hospitalization	22 (7.1)	14 (6.2)	8 (9.6)	0.3
Pregnancy or Puerperium *	14 (8.8)	9 (7.4)	5 (13.5)	0.32
Oral contraceptives *	40 (25.2)	32 (26.2)	8 (21.6)	0.57
Comorbidities, n (%)				
Arterial hypertension	90 (29.1)	65 (28.8)	25 (30.1)	0.82
Diabetes	13 (4.2)	10 (4.4)	3 (3.6)	1.00
COPD	16 (5.2)	13 (5.8)	3 (3.6)	0.57
Heart failure	9 (2.9)	7 (3.1)	2 (2.4)	1.00
Therapy, n (%)				
Aspirin †	42 (13.6)	33 (14.6)	9 (10.8)	0.39
Sulodexide †	37 (12)	26 (11.5)	11 (13.3)	0.67
ACEI	50 (16.2)	35 (15.5)	15 (18.1)	0.58
β-blockers	10 (3.2)	7 (3.1)	3 (3.6)	0.73
Statins	137 (44.3)	99 (43.8)	38 (45.8)	0.76
Laboratory investigations				
Creatinine, μmol L^{-1}	70 (61.9-79.6)	70 (61-79.6)	70.7 (62-79.6)	0.31
Glucose, mmol L^{-1}	5 (4.7-5.5)	5 (4.7-5.5)	5.1 (4.8-5.5)	0.47
TG, mmol L^{-1}	1.2 (0.8-1.7)	1.17 (0.8-1.7)	1.3 (0.9-1.9)	0.04

TC, mmol L^{-1}	5.2 (4.4-5.8)	5.2 (4.3-5.8)	5.3 (4.8-6)	0.13
LDL-C, mmol L^{-1}	3.1 (2.6-3.8)	3.0 (2.5-3.7)	3.2 (2.8-4)	0.06
HDL-C, mmol L^{-1}	1.5 (1.2-1.7)	1.5 (1.2-1.7)	1.4 (1.1-1.7)	0.25

Values are given as mean ± standard deviation (SD), median (interquartile range) or number (percentage).
PTS, post-thrombotic syndrome; COPD, chronic obstructive pulmonary disease; ACEI, angiotensin-converting-enzyme inhibitor; TG, triglycerides; TC, total cholesterol; LDL-C, low-density lipoprotein cholesterol; HDL-C, high-density lipoprotein cholesterol. *Females only.
†Medications initiated after anticoagulation withdrawal during follow-up.

Supplementary Table 2. Characteristics of obese and non-obese patients.

Variable	Obese patients (n=63)	Non-obese patients (n=246)	P value
Age, years	48 (36-55)	46 (36-54)	0.59
Male sex, n (%)	30 (47.6)	120 (48.8)	0.87
BMI, kg m^{-2}	32 (30.6-33.9)	25.2 (22.9-27.2)	<0.0001
Duration of anticoagulation treatment, months	10 (8-13)	10 (7-12)	0.17
Clinical Characteristics, n (%)			
Unprovoked VTE	31 (49.2)	126 (51.2)	0.78
DVT alone	48 (76.2)	194 (78.9)	0.65
Localization of isolated DVT, n (%)			
proximal DVT	37 (58.7)	134 (54.5)	0.54
DVT distal	11 (17.5)	60 (24.4)	0.24
PE+DVT, n (%)	15 (23.8)	52 (21.1)	0.65
Family history of VTE, n (%)	11 (17.5)	35 (14.2)	0.52
Comorbidities, n (%)			
Arterial hypertension	25 (39.7)	65 (26.4)	0.04
Diabetes	7 (11.1)	6 (2.4)	0.01
COPD	2 (3.2)	14 (5.7)	0.54
Heart failure	0 (0)	9 (3.7)	0.21
Therapy, n (%)			

Variable	Obese patients (n=63)	Non-obese patients (n=246)	P value
Aspirin *	12 (19)	30 (12.2)	0.16
Sulodexide *	6 (9.5)	31 (12.6)	0.5
ACEI	14 (22.2)	36 (14.6)	0.14
β-blockers	4 (6.3)	6 (2.4)	0.12
Statins	19 (30.2)	118 (48)	0.01
Compression Therapy	42 (66.7)	169 (68.7)	0.76
Laboratory investigations			
Creatinine, µmol L^{-1}	70 (61-79)	70.4 (61.9-79.6)	0.64
Glucose, mmol L^{-1}	5.2 (4.8-5.8)	5 (4.7-5.4)	0.09
TG, mmol L^{-1}	1.8 (1.2-2.5)	1.1 (0.8-1.5)	<0.0001
TC, mmol L^{-1}	5.3 (1)	5.1 (1)	0.32
LDL-C, mmol L^{-1}	3.2 (2.7-4)	3 (2.5-3.8)	0.14
HDL-C, mmol L^{-1}	1.3 (1.1-1.6)	1.5 (1.2-1.7)	0.006
CRP, mg L^{-1}	1.5 (1-2.5)	1.5 (0.9-2.2)	0.71
INR	0.98 (0.9-1.02)	0.98 (0.9-1.05)	0.4
Fibrinogen, g L^{-1}	3.22 (2.60-4.10)	2.97 (2.51-3.81)	0.06
D-dimer, ng mL^{-1}	287 (227-347)	278 (223-336)	0.57
tPa, ng mL^{-1}	9.6 (7.2-11.5)	9.6 (7.1-11.4)	0.89
PAI-1, ng mL^{-1}	10.9 (8.3-15)	11.2 (8.5-14.7)	0.79
Peak thrombin, nM	254.6 (203-289.9)	244.3 (211-295)	0.7
Adipokines			
Adiponectin, µg mL^{-1}	13.9 (12.8-16.1)	15 (13.9-17.2)	<0.0001
Leptin, ng mL^{-1}	28 (26.6-30.4)	27.1 (23.5-30.3)	0.01
Resistin, pg mL^{-1}	14.7 (13.5-15.9)	14.9 (13.9-16.5) †	0.23

Values are given as mean ± standard deviation (SD), median (interquartile range) or number (percentage).
BMI, body mass index; VTE, venous thromboembolism; DVT, deep vein thrombosis; PE, pulmonary embolism; CRP, C-reactive protein; INR, international normalized ratio; tPa, tissue plasminogen activator; PAI-1, plasminogen activator inhibitor-1; for other abbreviations see Supplementary Table 1.* Medications initiated after anticoagulation withdrawal during follow-up. † Data unavailable for one patient.

Supplementary Table 3. The logistic regression model for risk factors of PTS with continuous BMI.

Variable	OR per	Univariate		Multivariable*	
		OR (95% CI)	P	OR (95% CI)	P
Age †	10 years	0.89 (0.72-1.10)	0.27		
Male †	No/Yes	1.46 (0.88-2.42)	0.14	1.81 (0.9-3.62)	0.1
BMI †	1 kg m^{-2}	1.11 (1.05-1.18)	0.0008	0.95 (0.86-1.06)	0.36
Duration of anticoagulation treatment	1 month	1.03 (0.96-1.10)	0.49		
Smoking	No/Yes	0.94 (0.56-1.60)	0.83		
Family history of VTE	No/Yes	1.56 (0.80-3.04)	0.19		
Unprovoked VTE	No/Yes	1.21 (0.73-2.00)	0.47		
Proximal DVT	No/Yes	1.22 (0.66-2.25)	0.53		
Recurrent DVT ipsilateral	No/Yes	1.51 (0.58-3.92)	0.4		
Compression therapy	No/Yes	0.76 (0.45-1.30)	0.31		
Aspirin ‡	No/Yes	0.71 (0.33-1.56)	0.39		
Sulodexide ‡	No/Yes	1.18 (0.55-2.50)	0.68		
Statins	No/Yes	1.08 (0.65-1.80)	0.76		
Creatinine	1 µmol L^{-1}	1.01 (0.99-1.03)	0.22		
Glucose	1 mmol L^{-1}	1.05 (0.73-1.52)	0.79		
Triglycerides	1 mmol L^{-1}	1.36 (0.94-1.97)	0.11		
CRP	1 mg L^{-1}	1.07 (0.90-1.27)	0.43		
Fibrinogen	1 g L^{-1}	1.08 (0.82-1.42)	0.59		
D-dimer	100 ng mL^{-1}	1.01 (0.78-1.31)	0.91		
tPa †	1 ng mL^{-1}	1.11 (1.02-1.21)	0.02	1.06 (0.95-1.2)	0.3
PAI-1	1 ng mL^{-1}	1.04 (0.99-1.08)	0.08		
Peak thrombin	10 nM	0.99 (0.96-1.03)	0.77		
Adiponectin	1 µg mL^{-1}	0.52 (0.43-0.64)	<0.001	0.42 (0.31-0.57)	<0.0001
Leptin	1 ng mL^{-1}	1.41 (1.28-1.56)	<0.001	1.49 (1.31-1.69)	<0.0001
Resistin	1 pg mL^{-1}	1.02 (0.92-1.12)	0.75		

PTS, post-thrombotic syndrome; OR, odds ratio; CI, confidence intervals; other abbreviations see Supplementary Table 1 and Supplementary Table 2. *Multivariable model was fitted using backward stepwise regression. Adjusted for age. †Variable locked in the model. ‡Medications initiated after anticoagulation withdrawal during follow-up.

Supplementary Table 4. The obesity-stratified logistic regression analysis for risk factors of PTS.

Variable	OR per	OR (95% CI)	p
Male	No/Yes	1.84 (0.95-3.56)	0.07
tPa	1 ng mL^{-1}	1.06 (0.94-1.18)	0.33
Adiponectin	1µg mL^{-1}	0.49 (0.38-0.64)	<0.0001
Leptin	1 ng mL^{-1}	1.41 (1.25-1.58)	<0.0001

For abbreviations see Supplementary Table 1 and Supplementary Table 2. * Model fitted on the basis of multivariable model presented in Table 2. Adjusted for age.

Supplementary Table 5. The logistic regression model for categorical risk factors of PTS.

Variable	OR per	Univariate		Multivariable*	
		OR (95% CI)	P	OR (95% CI)	P
Age †	10 years	0.89 (0.72-1.10)	0.27		
Male †	No/Yes	1.46 (0.88-2.42)	0.14	1.29 (0.72-2.33)	0.39
Obesity †	No/Yes	1.35 (0.74-2.47)	0.33	0.87 (0.4-1.88)	0.71
Duration of anticoagulation treatment	1 month	1.03 (0.96-1.10)	0.49		
Smoking	No/Yes	0.94 (0.56-1.60)	0.83		
Unprovoked VTE	No/Yes	1.21 (0.73-2.00)	0.47		
Proximal DVT	No/Yes	1.22 (0.66-2.25)	0.53		
Recurrent DVT ipsilateral	No/Yes	1.51 (0.58-3.92)	0.4		
Family history of VTE	No/Yes	1.56 (0.80-3.04)	0.19	1.68 (0.74-3.77)	0.21
Aspirin ‡	No/Yes	0.71 (0.33-1.56)	0.39		
Sulodexide ‡	No/Yes	1.18 (0.55-2.50)	0.68		

Statins	No/Yes	1.08 (0.65-1.80)	0.76		
Compression therapy	No/Yes	0.76 (0.45-1.30)	0.31		
Creatinine	1 µmol L^{-1}	1.01(0.99-1.03)	0.22		
Glucose	1 mmol L^{-1}	1.05 (0.73-1.52)	0.79		
Triglycerides	1 mmol L^{-1}	1.36 (0.94-1.97)	0.11		
CRP	1 mg L^{-1}	1.07 (0.90-1.27)	0.43		
Fibrinogen	1 g L^{-1}	1.08 (0.82-1.42)	0.59		
D-dimer	100 ng mL^{-1}	1.01 (0.78-1.31)	0.91		
tPa †	1 ng mL^{-1}	1.11 (1.02-1.21)	0.02		
PAI-1	1 ng mL^{-1}	1.04 (0.99-1.08)	0.08		
Peak thrombin	10 nM	0.99 (0.96-1.03)	0.77		
Adiponectin	≤12.8 vs >12.8 µg mL^{-1}	0.1 (0.04-0.22)	<0.0001	0.08 (0.03-0.22)	<0.0001
Leptin	≤32.8 vs >32.8 ng mL^{-1}	8.42 (3.92-18.13)	<0.0001	6.51 (2.8-15.14)	<0.0001
Resistin	1 pg mL^{-1}	1.02 (0.92-1.12)	0.75		

OR, odds ratio; CI, confidence intervals; for other abbreviations see Supplementary Table 1 and Supplementary Table 2. *Multivariable model was fitted using backward stepwise regression. Adjusted for age and tPa. †Variable locked in the model. ‡Medications initiated after anticoagulation withdrawal during follow-up.

Supplementary Table 6. Recurrences of venous thromboembolism during 2-year follow up.

Variables, n (%)	VTE patients (n=309)	No PTS (n=226)	PTS (n=83)	P value
DVT recurrence	35 (11.3)	23 (10.2)	12 (14.5)	0.29
DVT recurrence in the same localization (in the reference to patients with DVT recurrence)	20 (57.1)	13 (56.5)	7 (58.3)	0.92
Unprovoked recurrence (in the reference to patients with DVT recurrence)	20 (57.1)	12 (52.2)	8 (66.7)	0.41
PE recurrence	10 (3.2)	7 (3.10)	3 (3.6)	0.73
VTE recurrence	35 (11.3)	23 (10.2)	12 (14.5)	0.29
DVT recurrence in the same localization	20 (6.5)	13 (5.8)	7 (8.4)	0.92
Unprovoked recurrence	20 (6.5)	12 (5.3)	8 (9.6)	0.34

For other abbreviations see Supplementary Table 1 and Supplementary Table 2.
